# Omics data integration identifies *ELOVL7* and *MMD* gene regions as novel loci for adalimumab response in patients with Crohn’s disease

**DOI:** 10.1038/s41598-021-84909-z

**Published:** 2021-03-09

**Authors:** Mario Gorenjak, Mateja Zupin, Gregor Jezernik, Pavel Skok, Uroš Potočnik

**Affiliations:** 1 Centre for Human Molecular Genetics and Pharmacogenomics, Faculty of Medicine, University of Maribor, Taborska ulica 8, 2000 Maribor, Slovenia; 2 Department of Gastroenterology, University Clinical Centre Maribor, Ljubljanska ulica 5, 2000 Maribor, Slovenia; 3 Laboratory of Biochemistry, Molecular Biology and Genomics, Faculty of Chemistry and Chemical Engineering, University of Maribor, Smetanova 17, 2000 Maribor, Slovenia

**Keywords:** Genetics, Gene expression, Gene regulation, Genetic association study, Genetic markers, Genomics, Medical genetics, Sequencing

## Abstract

Response to anti-TNF therapy is of pivotal importance in patients with Crohn’s disease (CD). Here we integrated our and previously reported PBMC derived transcriptomic and genomic data for identification of biomarkers for discrimination between responders and non-responders to anti-TNF therapy. CD patients, who were naïve with respect to the treatment with biologicals, were enrolled in the study. DNA and RNA were extracted from peripheral blood mononuclear cells. RNA-seq was performed using BGISEQ-500. Genotyping was performed using Infinium Global Screening Array. Association regressions were carried out with 12 week response to adalimumab as an outcome variable. RNA-seq analysis confirmed 7 out of 65 previously suggested genes involved in anti-TNF response. Subsequently, analysis of single nucleotide variants in regions of confirmed genes identified 5 variants near *MMD* and two in *ELOVL7* intronic regions associated with treatment response to anti-TNF. Functional analysis has shown that rs1465352, rs4422035 and rs78620886 are listed at H3K9ac_Pro histone modification epigenetic mark. The present study confirmed MMD and ELOVL7 involvement in anti-TNF response and revealed that the regulation of *MMD* and *ELOVL7* gene regions in ADA response may be a part of a complex interplay extending from genetic to epigenetic and to transcriptomic level.

## Introduction

Inflammatory bowel disease (IBD) includes both Crohn's disease (CD) and ulcerative colitis (UC) and results from improper inflammatory response to microbiota in a host with genetic susceptibility^1^. With tumor necrosis factor alpha (TNF-α) being the key cytokine in inflammation, many anti-TNF therapies emerged and dominated in the treatment of CD^2,3^. On one hand, disease management was significantly improved with anti-TNF agents, such as adalimumab (ADA) and infliximab (IFX)^4,5^, but on the other hand, 30% of patients do not respond to the anti-TNF treatment and of those who initially benefit, up to 40% lose their response later^2,4,6,7^. Moreover, unnecessary continuation of the anti-TNF therapy may severely impede patients' quality of life and impose adverse effect risk without clinical justification with emerging of an additional challenge of loss of response management^8,9^. Thus, identification of anti-TNF therapy response predictors is of pivotal importance and is posing an additionally challenge in the last decade. Prediction of response is also needed as we now have other therapies with different targets available^10^. Several studies have focused on genetic background and gene expression^11–17^ as potential biomarkers for anti-TNF treatment response. Currently the genomics markers of anti-TNF response in CD are not reaching significant thresholds and practically no reproducibility between genetic and expression markers exists, as identified by the first integration of genomic and expression data prior to anti-TNF treatment in a systematic review^18^. Prediction profiles have been mostly identified using epithelial gene expression data for which colonoscopy and biopsy sampling are needed^16,17^. However, obtaining colon biopsies is a highly invasive procedure. Studies which would focus on non-invasive genetic expression biomarkers, such as gene expression in peripheral blood mononuclear cells (PBMC) for anti-TNF response identification are scarce and non-homogenous in terms of anti-TNF therapy and disease classification.

Therefore, the aim of the present study is to integrate transcriptomic and genomic data obtained from blood of CD patients, who were naïve with respect to the treatment with biologicals, using our and previously reported peripheral blood derived transcriptomic data and using a modified contemporary combination of omics approach as described previously^19^. For that, we integrated our omics data based on 46 genes identified in CD and rheumatoid arthritis (RA) cohorts, which appeared the most significant to differentiate responders from non-responders to anti-TNF therapy (Table 1)^20^. We also included four genes that correlated with changes in endoscopic activity in UC patients with anti-TNF therapy and three differentially expressed genes between responding and non-responding IBD patients to anti-TNF therapy as identified by a cell-centered meta-analysis (Table 1)^21,22^. Additionally we included 12 independently confirmed and previously integrated genomic and expression (RNA and protein) markers of anti-TNF therapy response in CD patients (Table 1)^18^. The investigation gene panel built for the present study was altogether based on 65 previously suggested candidate genes in 2126 individuals with IBD and 34 individuals with RA, and treated with anti-TNF therapy.

**Table 1:** Previously identified anti-TNF response genes in PBMC and integrated genomic markers.

Study (N)	Phenotype	Genes					
Mesko et al.^20^ (20CD + 19 RA) replication: (20 CD + 15 RA)	CD	*UBE2EH*	*ODC1*	*CD300E*	*PCYT1B*	*AIDA*	*RIOK3*
		*PBX1*	*ARHGEF12*	*MMD*	*BMP6*	*WARS*	*ENDOD1*
		*CYP1B1*	*GCLC*	*BTN3A2*	*RNF11*	*CADM2*	*MAP1LC3B*
		*KAT2B*	*IL18R1*	*FMN1*	*CA2*	*IL1RL1*	
	RA	*IFI44*	*PF4*	*RAVER2*	*IFIT1*	*IFIT3*	*APOBEC3A*
		*ELOVL7*	*MICA*	*OR2A9P*	*IFI44L*	*MME*	*CCL4*
		*RGS1*	*IRF7*	*EPSTI1*	*MX1*	*DHRS9*	*RFC1*
		*SERPING1*	*IFI6*	*IFI35*	*IFITM1*	*GZMB*	
Gaujoux et al.^22^ (46 UC + 19 CD)	IBD/IDBu	*TREM1*	*CCR2*	*CCL7*			
Planell et al.^21^ (136 UC)	UC	*HP*	*CD177*	*GPR84*	*S100A12*		

Study (N)	Tissue	Genes					
Gole et al.^18^ (1885 CD)	Colon/mucosa/stool	*ACSL6*	*CASP9*	*FCGR2C*	*HSPA7*	*S100A8*	*S100A9*
		*SLC22A4*	*SLC22A5*	*CCHCR1*			
	Blood/PBMCs/ serum	*CRP*	*RPS23P10*	*SLC22*			

*CD* Crohn's disease, *RA* rheumatoid arthritis, *IBD* inflammatory bowel disease, *IBDu* inflammatory bowel disease unclassified, *UC* ulcerative colitis.

## Results

### RNA-seq analysis

**Figure 1:**
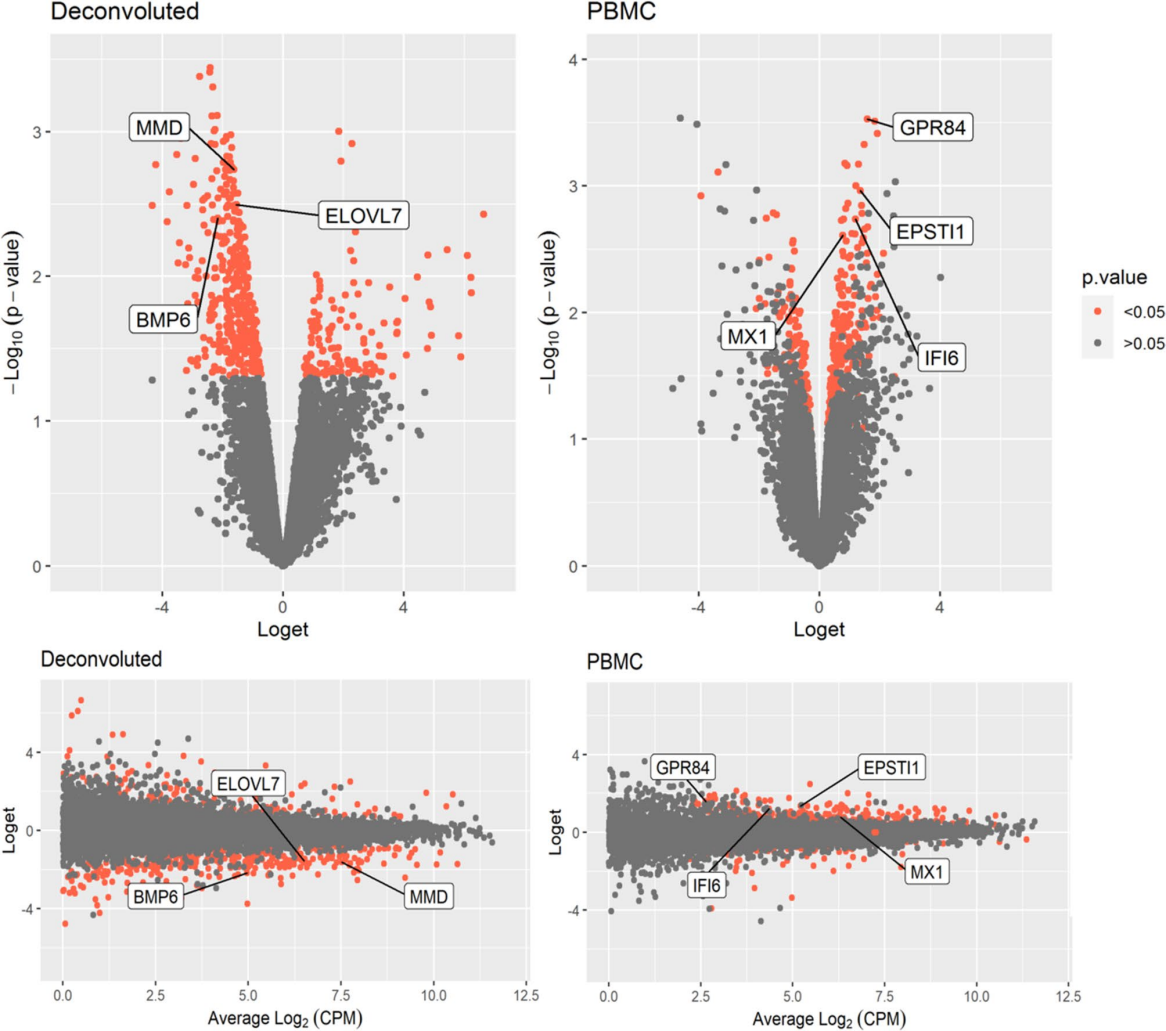
RNA-seq volcano and smear plots of RNA-seq analyses. RNA-seq analysis. The plots were constructed using *ggplot2* R package^23^.

Using our RNA sequencing data we performed an expression study using previously suggested candidate genes discriminating anti-TNF response in CD patients. For that, we used two different approaches. In first approach, the analysis was adjusted to deconvoluted fractions of PBMCs and has identified 11 differentially expressed genes out of which *MMD*, *ELVOL7* and *BMP6* remained significantly down-regulated in responders relative to non-responders after correction (Table 2, Fig. 1). In the second approach, analysis was carried out unadjusted in order to obtain PBMC panel. The analysis has confirmed 10 differentially expressed genes out of which 4 (*GPR84*, *EPSTI1*, *IFI6*, *MX1*) remained significantly up-regulated after correction for multiple comparison in responders relative to non-responders (Table 2, Fig. 1).

**Table 2:** Confirmation of candidate genes previously associated with anti-TNF response using RNA-seq analysis.

	Entrez ID	Symbol	Loget	Loget 95CI L	Loget 95CI U	*p*-value	Adj. *p-*value
Deconvoluted	**23531**	***MMD***	**− 1.62**	**− 2.32**	**− 0.93**	**0.0018**	**0.0201**
	**79993**	***ELOVL7***	**− 1.55**	**− 2.30**	**− 0.80**	**0.0032**	**0.0351**
	**654**	***BMP6***	**− 2.17**	**− 3.28**	**− 1.06**	**0.0040**	**0.0436**
	57126	*CD177*	**− **2.68	**− **4.29	**− **1.07	0.0078	0.0861
	94240	*EPSTI1*	1.19	0.25	2.13	0.0226	0.2482
	53831	*GPR84*	1.60	0.33	2.87	0.0228	0.2507
	4953	*ODC1*	**− **1.40	**− **2.57	**− **0.24	0.0269	0.2957
	23052	*ENDOD1*	**− **1.57	**− **2.91	**− **0.24	0.0292	0.3211
	3437	*IFIT3*	0.91	0.05	1.78	0.0425	0.4673
	2537	*IFI6*	1.07	0.03	2.12	0.0463	0.5093
	5996	*RGS1*	2.17	0.05	4.29	0.0465	0.5119
PBMC	**53831**	***GPR84***	**1.59**	**1.00**	**2.17**	**0.0003**	**0.0030**
	**94240**	***EPSTI1***	**1.36**	**0.74**	**1.97**	**0.0011**	**0.0109**
	**2537**	***IFI6***	**1.20**	**0.61**	**1.79**	**0.0018**	**0.0182**
	**4599**	***MX1***	**0.78**	**0.37**	**1.18**	**0.0025**	**0.0245**
	10561	*IFI44*	0.80	0.25	1.35	0.0107	0.1070
	10964	*IFI44L*	1.35	0.38	2.32	0.0128	0.1281
	710	*SERPING1*	1.35	0.18	2.52	0.0294	0.2940
	3430	*IFI35*	0.61	0.07	1.16	0.0316	0.3157
	3665	*IRF7*	0.46	0.04	0.87	0.0342	0.3422
	3434	*IFIT1*	0.90	0.02	1.78	0.0452	0.4521

*Loget* Log_2_FC Responders relative to non-responders, *95 CI* 95% confidence interval, *L* lower, *U* upper, Bold text indicates statistically significant genes after adjustment.

### Integration to genomics

**Figure 2:**
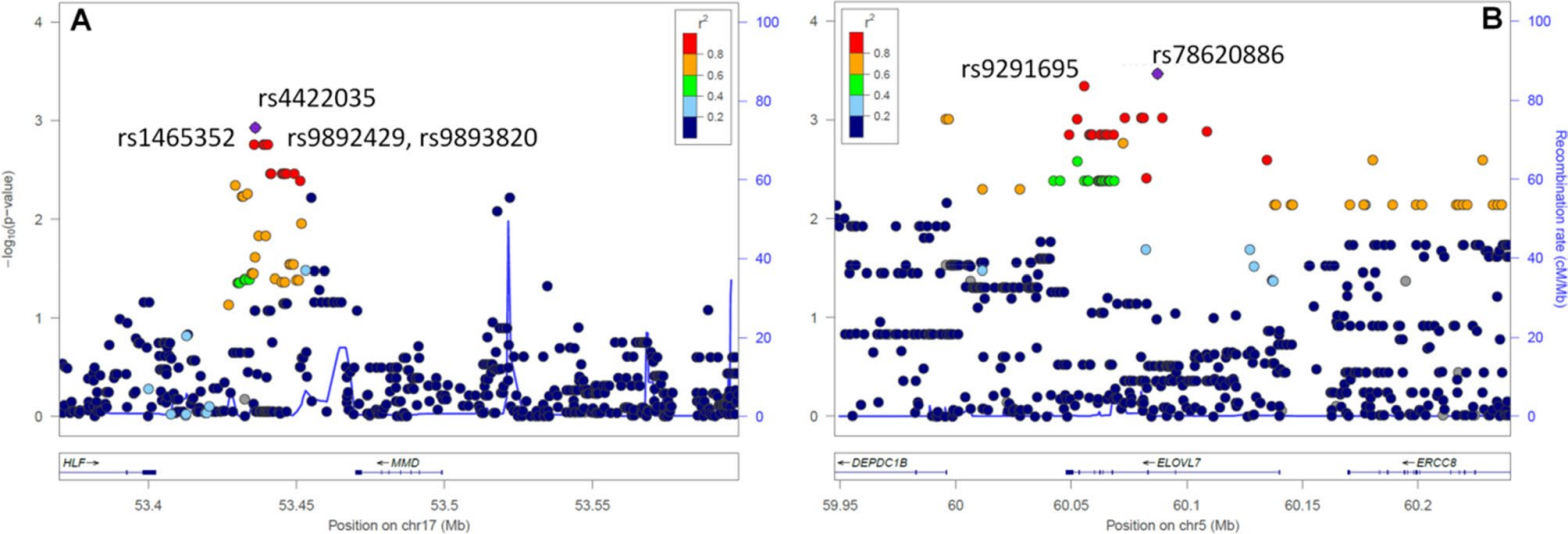
Regional Manhattan plots for *MMD* and *ELOVL7* ± 100 kb gene region. (**A**) *MMD*; (**B**) *ELOVL7.* Regional Manhattan plots were constructed using LocusZoom^24^.

Using an integrative transcriptomic-genomic approach we analyzed single nucleotide variants ranging ± 100 kb from previously identified differentially expressed genes in 84 patients with CD. Association analysis has identified 5 statistically significant variants near gene *MMD* (Fig. 2A) and 2 statistically significant variants in *ELOVL7* gene region (Fig. 2B). For single nucleotide polymorphisms (SNPs) rs1465352, rs4422035, rs9892429, rs9893820 and rs11656799 near gene *MMD* the results have shown significant higher alternative allele frequency in non-responders in comparison to responders (Table 3). For the most significant rs4422035 the analysis has shown higher alternative allele frequencies in non-responders (Allele C: 0.69) in comparison to responders (Allele C: 0.45) (p = 0.018; OR 0.22; CI 0.09–0.55). Additional LD assessment has shown that that rs4422035 and rs1465352 are in linkage disequilibrium (D′: 1.0; r^2^: 0.87; p < 10^–4^). Identified rs9892429, rs9893820 and rs11656799 have shown higher linkage disequilibrium with rs1465352 (D′: 1.0; r^2^: 0.96; p < 10^–4^) as in comparison to most significant rs4422035, which is also evident from the Table 3. Similar effect was observed also for variants identified in *ELOVL7* gene region. Both identified variants have shown the same trend of alternative allele frequencies (Table 3). For the most significant rs78620886 the analysis has shown higher alternative allele frequencies in non-responders (Allele A: 0.26) in comparison to responders (Allele A: 0.03) (p = 0.021; OR 0.05; CI 0.01–0.27). Additional LD analysis has also shown that rs78620886 is in linkage disequilibrium with rs9291695 (D′: 1.0; r^2^: 0.95; p < 10^–4^).

**Table 3:** SNPs significantly associated with anti-TNF response and located near genes differentially expressed in responders and non-responders according to our RNA-seq results.

SNP	Chr	Position	Alleles	OR	95 CI L	95 CI U	AF R	AF NR	*p*-value	Adj. *p*-value
rs1465352	17	53435734	T/C	0.25	0.11	0.60	0.42	0.66	0.0017	0.0261
rs4422035	17	53436116	A/C	0.22	0.09	0.55	0.45	0.69	0.0012	0.0184
rs9892429	17	53438909	G/T	0.25	0.11	0.60	0.42	0.66	0.0017	0.0261
rs9893820	17	53439439	G/A	0.25	0.11	0.60	0.42	0.66	0.0017	0.0261
rs11656766	17	53440395	T/C	0.25	0.11	0.60	0.42	0.66	0.0017	0.0261
rs9291695	5	60055644	T/A	0.07	0.02	0.32	0.04	0.26	0.0005	0.0353
rs78620886	5	60087336	G/A	0.05	0.01	0.27	0.03	0.26	0.0003	0.0212

*OR* calculated for alternative allele and response, *95 CI* 95% confidence interval, *L* lower, *U* upper, *AF R* alternative allele frequency responders, *AF NR* alternative allele frequency non-responders.

### Functional analysis of identified variants

**Figure 3:**
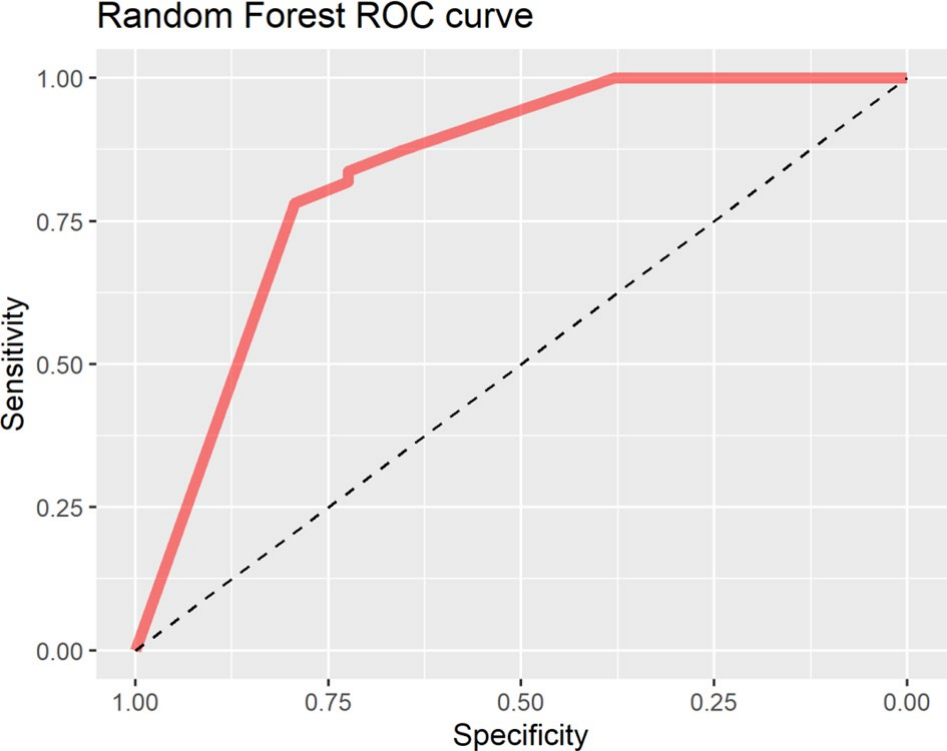
ROC curve of random forest classification of rs1465352, rs4422035 and rs78620886 genotypes for adalimumab response prediction. The ROC curve plot was constructed using *ggplot2* R package^23^.

Identified variants were further assessed for possible functional effects. All seven variants correspond to locations on non-coding DNA region. SNPs rs1465352 and rs4422035 near gene *MMD* were listed at H3K9ac_Pro histone acetylation mark in monocytes. Also the *ELOVL7* intronic rs78620886 was listed at the H3K9ac_Pro mark in primary T helper naive cells derived from peripheral blood (Table 4). Subsequently, eQTLs were also assessed (Table 4). For rs1465352 and rs11656766 significant eQTLs with *MMD* were listed in monocytes. For rs4422035, rs9892429 and rs9893820 no eQTL data was available in monocytes. For rs9291695 and rs78620886, significant eQTLs with *ELOVL7* were listed in EBV transformed lymphocytes. Additionally, genotyping results from rs1465352, rs4422035 and rs78620886 were used as variables for prediction value analysis using random forest classification and ROC analysis. Obtained results further confirmed the association of rs1465352, rs4422035 and rs78620886 in adalimumab response (AUC 0.833; CI 0.740–0.926) with sensitivity of 0.84, specificity of 0.72 and Youden index of 0.56 at the best cut-off point (Fig. 3).

**Table 4:** Functional analysis of significant SNPs.

SNP	HaploReg		eQTL			
	Epigenetics	Cells	Gene	Tissue	*p*-value	NES
rs1465352 (near gene 3′)	H3K9ac_Pro	Monocytes (RO 01746)	*MMD*	EBV Lymphocytes	0.2	0.093
				Whole blood	0.4	− 0.018
				Monocytes	**0.028**	**0.003**
rs4422035 (near gene 3′)	H3K9ac_Pro	Monocytes (RO 01746)	*MMD*	EBV Lymphocytes	0.7	0.025
				Whole blood	0.8	− 0.005
				Monocytes	NA	NA
rs9892429 (near gene 3′)	NA	NA	*MMD*	EBV Lymphocytes	0.3	0.07
				Whole blood	0.8	− 0.006
				Monocytes	NA	NA
rs9893820 (near gene 3′)	NA	NA	*MMD*	EBV Lymphocytes	0.3	0.071
				Whole blood	0.8	− 0.005
				Monocytes	NA	NA
rs11656766 (near gene 3′)	NA	NA	*MMD*	EBV Lymphocytes	0.3	0.071
				Whole blood	0.8	− 0.005
				Monocytes	**0.028**	**0.003**
rs9291695 (intron)	NA	NA	*ELOVL7*	EBV Lymphocytes	**0.01**	**0.454**
				Whole blood	0.2	0.044
rs78620886 (intron)	H3K9ac_Pro	Th0 (PBMC)	*ELOVL7*	EBV Lymphocytes	**0.02**	**0.553**
				Whole blood	0.1	0.062

*NES* Normalized effect size, *NA* not applicable, Bold text indicates statistically significant eQTLs.

### Mendelian randomization analysis

An additional Inverse-variance random-effect Mendelian Randomization (MR) analysis was performed. First, variants ranging ± 100 kb from *MMD* and *ELOVL7* were included into conditional regressions in order to detect independent signals conditioned to the identified most significant SNPs. Conditioning results have identified statistically significant rs12943795 besides rs4422035 for *MMD*, and rs182724486 and rs149880548 besides rs78620886 for *ELOVL7*. Statistically significant conditioned independent variants were further assessed for eQTLs in corresponding tissues/cells in which chromatin state assignment was listed in HaploReg and were included into subsequent MR analysis. MR analysis has proven the association of *ELOVL7* with response to adalimumab (p = 0.046; SE 2.812; CI95 11.13 to − 0.11). For *MMD* no MR analysis was performed due to unavailable eQTLs for independent rs12943795 and rs4422035 SNPs in corresponding tissues/cells.

### Validation of genes using RT-qPCR

**Figure 4:**
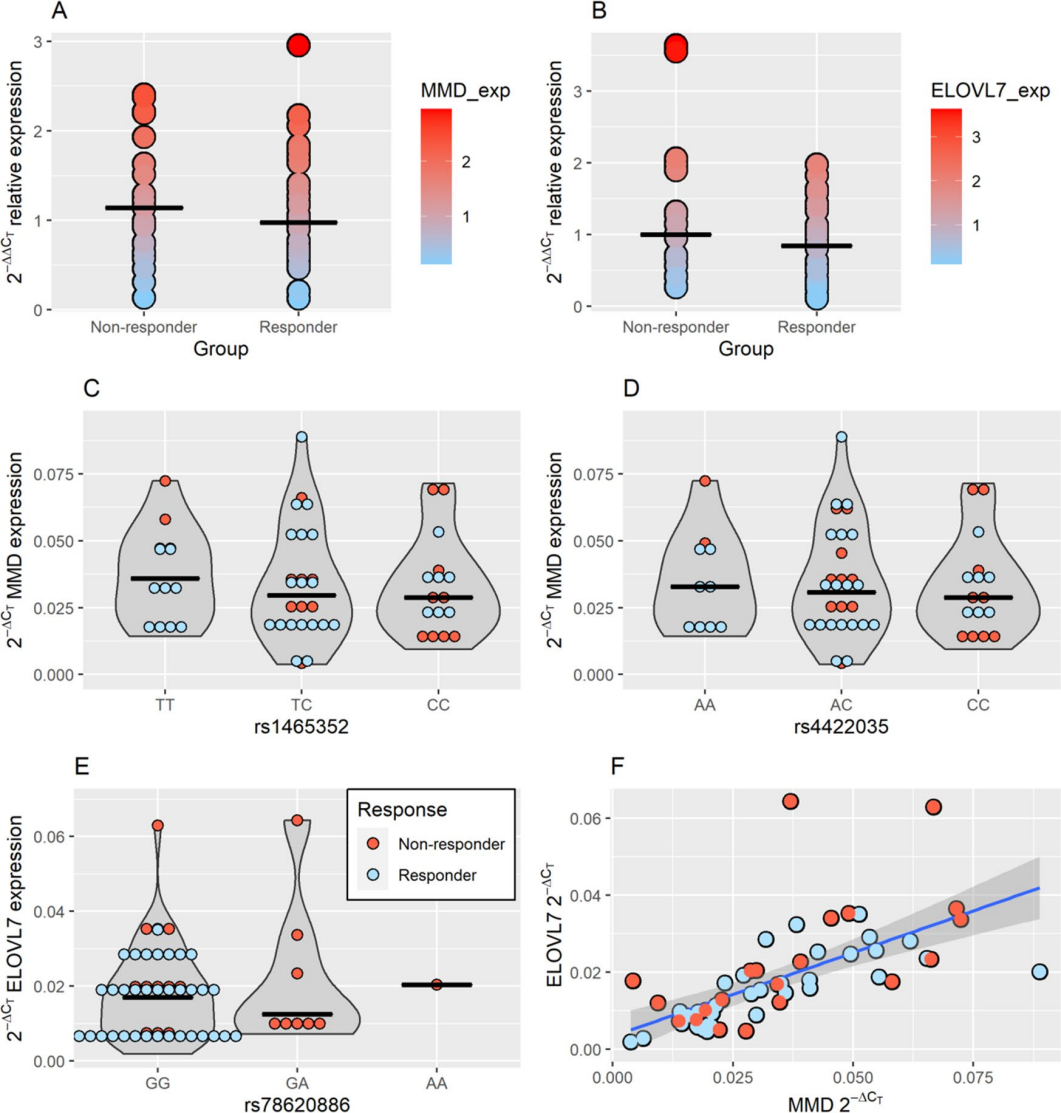
Validation of results using RT-qPCR, eQTL analysis and correlation estimation. (**A**) *MMD* relative expression; (**B**) *ELOVL7* relative expression; (**C**) eQTL for rs1465352 and *MMD*; (**D**) eQTL for rs4422035 and *MMD*; (**E**) eQTL for rs78620886 and *ELOVL7*; (**F**) Correlation between *MMD* and *ELOVL7* expression. Black lines represent median expression. Blue line represents correlation regression curve with standard error. The plots were constructed using *ggplot2* R package^23^.

In order to assess and validate the findings of RNA-seq analysis and subsequent genomic variants, RT-qPCR was performed. First, reference genes were checked for stability between responders and non-responders. Both genes, *ACTB* (p = 0.768) and *B2M* (p = 0.782) proved to be stably expressed in both groups. Subsequently gene expression of *MMD* and *ELOVL7* was measured in PBMCs. Both genes were down-regulated in responders, but statistical significance was observed only at *ELOVL7*, which was 1.43-fold down-regulated in responders relative to non-responders (p = 0.016; β − 0.535; SE 0.214) (Fig. 4B). *MMD* was 1.10-fold down-regulated in responders relative to non-responders (p = 0.498; β − 0.141; SE 0.656) (Fig. 4A). Additionally eQTLs in PBMCs were assessed for SNPs where chromatin state assignment was listed. No statistically significant eQTLs were observed for rs1465352 (p = 0.564; β 0.002; SE 0.004) (Fig. 4C), rs4422035 (p = 0.752; β 0.001; SE 0.004) (Fig. 4D) and rs78620886 (p = 0.448; β 0.003; SE 0.004) (Fig. 4E). In order to check the interplay of *MMD* and *ELOVL7* a correlation analysis of expression was performed. Correlation analysis revealed a strong correlation between genes (p = 2.57 × 10^–13^; ρ = 0.799) (Fig. 4F).

## Discussion

Anti-TNF therapy is of pivotal importance in patients with CD and genomic biomarkers that would tailor personalized treatment are currently needed, in particular as new biologicals for CD treatment have recently emerged. In the recent systematic review of anti-TNF genomic biomarkers in CD, it was noted that almost no candidate gene could be confirmed in an independent association study and no overlap was detected among genes identified with association and gene expression studies^18^. In the present study, we have adopted a new approach^19^ for the integration of PBMCs derived transcriptomic and genomic data. To our knowledge, this is the first time deconvolution was applied in order to account for PBMC composition in anti-TNF response transcriptomics, which identified two novel genetic loci on chromosomes 17 near gene *MMD* and 5 in gene *ELOVL7*, associated with anti-TNF response in CD patients.

For that, we included patients with CD, who were naïve with respect to the treatment with biologicals and who were subsequently treated with ADA and investigated PBMC derived transcriptomic-genomic data integration with targeting on previously identified gene expression panels for differentiation of responders from non-responders to anti-TNF therapy. Additionally, we investigated independently confirmed and previously integrated genomic and expression (RNA and protein) markers of anti-TNF therapy response in CD patients. Thus, the targeted expression panel consisted altogether of 65 genes, which were previously identified in 2126 individuals with IBD and 34 individuals with RA as potential predictors for different anti-TNF therapy (ADA, IFX, golimumab) responses in peripheral blood of CD, UC, unclassified IBD or RA patients^18,20–22^.

First, using RNA-seq and using 3 responders and 3 non-responders to ADA we confirmed differentially expressed genes from previously identified gene panel. The model fitting for estimation of differentially expressed genes was carried out in two different ways—adjusted to deconvolution results and not adjusted in order to obtain PBMC expression picture. The results have shown that in deconvoluted analysis *MMD*, *ELOVL7* and *BMP6* remained significantly differentially expressed after correction for multiple comparisons. All three genes were down-regulated in responders relative to non-responders (up-regulated in non-responders relative to responders). Using unadjusted model fitting we observed the confirmation of *GPR84*, *EPSTI1*, *IFI6*, *MX1*, which remained significantly differentially expressed after the application of multiple comparison correction. All four were found to be up-regulated in responders relative to non-responders (down-regulated in non-responders relative to responders). Subsequently, confirmed genes were further analyzed using integration with genomic data obtained from 84 CD patients, who were naïve with respect to the treatment with biologicals, using a genotype microarray and using an association analysis with 12 week response as an outcome variable. Imputed high quality single nucleotide variants were analyzed using a margin of ± 100 kb from confirmed genes in RNA-seq analysis in order to include variants in regulatory gene regions. The results have identified two significant signals, one near gene *MMD* and one in *ELOVL7* gene region after application of Bonferroni-like correction using empirical autocorrelation estimation, but no statistically significant signals were observed in other 5 genes. Both *MMD* and *ELOVL7* were identified in deconvoluted approach. Thus, obtained results are pointing to increased robustness of analysis when using deconvolution of PBMCs. As previously shown using deconvolution-meta-analysis paradigm, adjustment to estimation of cell proportions can capture differential expression otherwise masked due to cellular composition variation^22^. Expression of *MMD* (Monocyte to macrophage differentiation-associated) gene can be detected in all tissues and its’ up-regulation is observed upon monocyte differentiation, which modulates the production of TNF-α and nitrous oxide (NO)^25^. TNF was previously identified among key players of the Schmitt et al. proposed model of anti-TNF non-response^26^ and was identified as a sole key player in the baseline response prediction screening^18^. It was also previously suggested that there is an association between the loss of anti-TNF response and ROS response^27^, which is in concordance with our findings as up-regulation of *MMD* gene modulates also the production of NO. *ELOVL7* (ELOVL Fatty acid elongase 7) gene is a member of 7 ELOVL isozymes which catalyze the first step of very long-chain fatty acid elongation cycle^28^. In the context of IBD, *ELOVL7* was previously associated only with protective association for hidradenitis suppurativa^29^. In aforementioned regions, the analysis identified SNPs rs1465352, rs4422035, rs9892429, rs9893820 and rs11656766 located downstream of *MMD* and SNPs rs9291695 and rs78620886 located in *ELOVL7* intronic regions. OR calculated for alternative allele for all SNPs indicates higher probability for non-responding to ADA therapy. To the best of our knowledge this is the first time that the aforementioned SNPs are associated with response to anti-TNF therapy in CD. Furthermore, functional analysis for *MMD* rs1465352 and rs4422035, and *ELOVL7* rs78620886 has shown that these SNPs are listed in HaploReg v4.1 at H3K9ac_Pro histone modification epigenetic mark in monocytes and lymphocytes, respectively. Histone modifications are implicated in influencing gene expression by establishing global chromatin environments and H3K9ac (Histone 3 Lysine 9 acetylation) histone modification is enriched in promoter regions containing critical regulatory elements necessary for transcription^30^. H3K9 is a residue that can be acetylated or methylated and H3K9ac was shown to be dramatically reduced during chromosome condensation^31^. Moreover, H3K9ac is associated with active promoters and is considered as a hallmark of active transcriptions^32^. As *MMD* and *ELOVL7* were both up-regulated in non-responders to ADA, we hypothesize that locations of rs1465352, rs4422035 and rs78620886 might play a significant role in histone modifications in terms of H3K9 residue acetylation and up-regulation of gene expression. Using ENSEMBL^33^ and GTExportal database^34^ statistically significant eQTLs with *MMD* and *ELOVL7* were also listed. Additionally, the implication of aforementioned SNPs in ADA response was also confirmed using random forest machine learning algorithm and ROC analysis, which has additionally confirmed the association of these three SNPs with ADA response in patients with CD. In order to fully address the implication of both genes in ADA response, a Mendelian randomization integrating eQTL and association analysis data was performed. Gene *ELOVL7* was subsequently again confirmed as a significant candidate, however no analysis was performed for *MMD* due to unavailability of eQTL data for selected variants in corresponding tissues/cells in used databases^33,34^. Furthermore, monocyte to macrophage differentiation was found to go along with profound changes in lipid-related transcriptome, leading to an induction of fatty-acid elongation^35^, which can elucidate the interplay of *MMD* and *ELOVL7* up-regulation in ADA non-responders. These findings are further supported by previously shown genetically influenced abnormalities in fatty acid profiles in IBD^36^. The main limitation of the present study is the limited sample size in transcriptome analysis. Nevertheless, we believe that using trimmed mean of M values method (TMM) normalization implemented in *edgeR* and the variance modeling at the observational level (VOOM) method implemented in the *limma* package before applying empirical Bayes over a linear model, made our analysis stringent enough to provide reliable results. The aforementioned concern was also addressed using RT-qPCR analysis for estimation of *MMD* and *ELOVL7* gene expression in PBMCs in a larger cohort in order to validate the findings. However, *MMD* and *ELOVL7* were identified using an approach with deconvolution of PBMCs, thus differences between responders and non-responders may not have been fully revealed using RT-qPCR. Obtained results have shown that both, *MMD* and *ELOVL7* tend to be up-regulated in non-responders relative to responders, but only *ELOVL7* reached statistical significance, which is consistent with RNA-seq findings. Furthermore, for SNPs where chromatin state assignment was listed, no trend of association with *MMD* and *ELOVL7* was observed in PBMCs, which is also consistent with reported eQTLs in whole blood. Moreover, using RT-qPCR we observed a strong correlation between *MMD* and *ELOVL7*, which further supports the interplay between these two genes and serves as additional supporting evidence of monocyte to macrophage differentiation and changes in lipid-related transcriptome. In addition, based on our results functional studies are warranted and could further unravel the interplay between *MMD*, *ELOVL7* and H3K9ac in response to anti-TNF therapy. We also acknowledge that the homogenous group of enrolled IBD classified CD patients and using only one anti-TNF agent represents the strength of our study design.

In summary, the present study integrated transcriptomic and genomic data obtained from CD patients’ PBMCs and identified two novel genetic loci, on chromosomes 17 and 5, associated with anti TNF response in CD patients and suggested *MMD* and *ELOVL7* as best candidates for involvement in anti-TNF response. Moreover, the present study revealed that the implication and regulation of *MMD* and *ELOVL7* gene regions in ADA response may be a part of a complex interplay extending from genetic to epigenetic and to transcriptomic level. This knowledge could be further translated into new clinical non-invasive baseline biomarkers for adalimumab response in patients with CD.

## Methods

All methods were carried out in accordance with relevant guidelines and regulations.

### Enrolled subjects

We included 84 Slovenian patients with CD who fulfilled the criteria for anti-TNF-α drug ADA (Humira, Abbott Laboratories, IL, USA), and who were naïve with respect to the treatment with biologicals. All enrolled subjects were of Slovenian (Caucasian—Central Europe) ethnicity. Baseline demographics of the patients are shown in Table 5. Inclusion criteria were refractoriness to corticosteroids, adverse effects to corticosteroids and lost response or intolerance to IFX as described previously^12^. Exclusion criteria were defined as presence of CD complications as stenosis, abscesses, total colectomy, history of murine proteins allergy, active tuberculosis or a serious infection in the last 3 months, pregnancy or lactation and malignancy^37^. First, a loading dose of 160 mg of ADA was administered, followed by 80 mg after 14 days and maintenance dose of 40 mg every other week. Azathioprine, 5-aminosalycylates, corticosteroids or antibiotic concomitant treatment was allowed if the dosage was stable in the last 3 months. The present study was evaluated and approved by Slovenian National Committee for Medical Ethics (KME 80/10/07, 21p/12/07). Written informed consent was obtained from all enrolled subjects. In case of minors, written informed consent was obtained from parents and/or legal guardians. Response to treatment was measured using IBD questionnaire (IBDQ) after 12 weeks of treatment. Response was defined as an increase in score > 22 points in comparison of pre-treatment score or total score > 170 points^38,39^.

**Table 5:** Baseline demographics of included patients.

	Responders	Non-responders	*p-*value
Gender (M/F)	22/33	11/18	1.000^c^
Age at Dg. (years)	27.2 ± 12.3	29.2 ± 12.0	0.366^m^
Smoking (Y/N)	31/24	18/11	0.649^c^
IBDQ (points)	155.0 ± 28.9	145.3 ± 27.0	0.090^m^
CDAI (points)	218.9 ± 137.5	265.2 ± 128.5	0.090^m^
Leukocytes (10^9^/L)	8.0 ± 2.8	8.5 ± 4.3	0.989^m^
Hemoglobin (g/L)	129.3 ± 21.4	120.3 ± 21.3	0.264^m^
CRP (mg/L)	21.4 ± 24.7	12.8 ± 11.5	0.531^m^

^c^Fisher exact test; ^m^Mann–Whitney U-Test.

### Extraction of nucleic acids

DNA and RNA were extracted from peripheral blood mononuclear cells (PBMC) using TRI-reagent (Merck, Darmstadt, Germany) according to manufacturer’s instructions. Purity and concentration of nucleic acids was determined using Synergy 2 spectrophotometer (Biotek, Winooski, VT, USA). Integrity of RNA was checked using agarose gel electrophoresis and 2100 Bioanalyzer Instrument (Agilent, Santa Clara, CA, USA) with RNA 6000 Nanochip.

### RNA-seq analysis

For RNA-seq analysis, 3 responders and 3 non-responders were selected. These patients were selected for RNA-seq based on available data for continuously measured treatment response, which was recorded at weeks 4, 12, 20 and 30. The selected 6 patients qualified as 3 were consistent responders and 3 consistent non-responders during all check point measurements. RNA RIN number was > 9.0 and 28S/18S ratio between 1.5 and 2.0 for all 6 samples. Both, lncRNA and mRNA 100 bp paired-end libraries were constructed using MGIEasy rRNA Depletion Kit (MGITech, Shenzen, China) and MGIEasy RNA Library Prep Set (MGITech) and using the BGISEQ-500 instrument (BGI, Hong Kong) at BGI facilities (BGI). Data analysis was performed using R 4.0.2 environment (R Core Team 2020, Vienna, Austria). Raw .fastq files were first assessed for quality using FastQC 0.11.9 software^40^. Technical sequences and sequencing adapters were trimmed using Trimmomatic 0.39 tool^41^. Paired-end reads were aligned to the hg19 reference genome using *Rsubread* 2.2.4 R package^42,43^. Mapped reads were counted and assigned to genomic features using *featureCounts*^44^ with requirement of both ends to be mapped. Counts per million (CPMs) were calculated using *edgeR* 3.30.3 R package^45^. Low expressed genes were filtered out based on CPMs corresponding to read counts of 10 and retained genes were normalized using the trimmed mean of M values method (TMM)^46^. Subsequently, mean–variance modeling at the observational level transformation (VOOM) was applied^47^. Differential expression of responders relative to non-responders was determined using linear models and empirical bayes implemented in *limma* 3.44.3 R package^48^ using two approaches. In first approach, the linear model was adjusted based on the deconvoluted peripheral blood mononuclear cell composition data. Deconvolution was used to obtain an estimation of the abundances of member cell types in a mixed cell population, using gene expression data in terms of proportions of different white cell subtypes in PBMCs^49^. Using raw counts, transcripts per million (TPM) were calculated, zero values were removed and data was deconvoluted using CIBERSORT^49^ and LM22^49^ signature matrix. Sample composition in terms of proportions of lymphocytes, monocytes/macrophages and neutrophils was used as a covariate in the model. Zero estimated cell proportions were excluded from analyses. In the second approach, the linear model was left unadjusted in order to obtain PBMC gene profile picture. Differential expression was considered for genes with adjusted *p*-value < 0.05.

### Association analysis

Samples obtained from 84 enrolled individuals were genotyped using Infinium Global Screening Array (GSA_24v1) (Illumina, San Diego, California, USA). Quality control of the genotyped data was performed as described previously^50^. Further steps of analysis were based on an integrative transcriptomic-genomic approach^19^. Subsequently, imputation was performed using Michigan Imputation Server Minimac3 genotype imputation algorithm and using HRC r1.1 2016 reference panel and SHAPEIT v2.r790 phasing^51^. Association between responders at week 12 vs. non-responders at week 12 was tested using binary logistic Wald test implemented in EPACTS 3.2.6^52^. Association analysis was adjusted to age at diagnosis, sex, azathioprine use, use of aminosalycylates, use of corticosteroids and first four principal components, which were calculated using PLINK 1.9 software^53,54^. Appropriate number of principal components was determined beforehand using *gap* v1.2.2 R package. Retained were only variants with imputation score (Rsq) > 0.3. Variants ± 100 kb from previously identified differentially expressed genes were further analyzed. Bonferroni-like correction was applied based on empirical autocorrelation determined using *coda* R package^55^. Statistically significant signal was considered for SNPs with adjusted *p*-value < 0.05. Linkage disequilibrium was calculated using LDlink software^56^.

### In silico functional analysis, prediction value estimation and visualization

Functional analyses were done using HaploReg v4.1^57^, GTExPortal^34^ and ENSEMBL^33^. Regional Manhattan plots were constructed using LocusZoom^24^. At SNPs, where chromatin state assignment was listed, predictive value of genotypes was further assessed using *randomForest* 4.6-14 R package ^58^ and Receiver Operating Characteristics analysis using *pROC* R package^59^. All other plots were constructed using *ggplot2* R package^23^.

### Mendelian randomization analysis

Data was further analyzed using Mendelian randomization integration of eQTL and association analysis data. Previously retained variants ± 100 kb from *MMD* and *ELOVL7* gene regions were further analyzed using GCTA-COJO conditional analysis^60^. GCTA-COJO conditional analysis was performed conditional on most significant SNP identified in previous association analysis. Reference dataset for GCTA-COJO was built based on previously imputed data. Variants with Rsq < 0.3 were removed from the reference dataset prior to analysis. Condition SNP and SNPs, which remained significant after conditioning, were included in subsequent Mendelian randomization analysis. Mendelian randomization was performed using *MendelianRandomization* v0.5.0 R package and inverse-variance weighted random-effect method^61^. Variant-gene association (eQTL) obtained from GTExportal^34^ and ENSEMBL^33^ in corresponding tissues/cells was included as exposure and variant-outcome association as outcome for MR analysis. If no eQTL data was available in corresponding tissues/cells, the variant was excluded from MR analysis.

### Validation of genes using RT-qPCR

A total of 1 µg of mRNA with RIN > 8 was obtained from 55 (21 non-responders and 34 responders) out of 84 enrolled individuals and was transcribed into cDNA using high capacity cDNA reverse transcription kit (Thermo Fisher, Waltham, MA, USA). mRNA nucleotide sequences of target genes *MMD* and *ELOVL7* were retrieved from NCBI Nucleotide database (www.ncbi.nlm.nih.gov/nuccore/). Primers for reference genes *ACTB* and *B2M* were obtained from previous study^17^. Isoform non-specific primers were hand-picked and designed using IDT OligoAnalyzer Tool (eu.idtdna.com/calc/analyzer). Primer sequences and accession numbers are summarized in Table 6. All primers were synthesized by Sigma (Merck, Darmstadt, Germany). Reverse transcription quantitative polymerase chain reaction (RT-qPCR) gene expression assay was carried out using Lightcycler 480 SYBR Green I Master Mix and Lightcycler 480 real time thermocycler (Roche, Basel, Switzerland) according to manufacturer’s instructions. 2 µL of 20-fold diluted cDNA (2.5 ng/µL) was used as a template. Primer efficiency was > 90% for all primer pairs. Melting curves for each sample were analyzed after each run in order to confirm specificity of amplification. Raw Ct values were obtained from three run-independent technical replicates for each sample. Normalization of raw data was performed using geometric averaging of reference genes and relative expression was calculated using 2^−ΔΔCt^ method^62^. Statistics was performed using linear 2^−ΔCt^ calculation and linear regression was adjusted to same covariates as aforementioned in association analysis. Stability of reference genes was statistically assessed using relative 2^−ΔCt^ calculation and Mann–Whitney U-Test. eQTLs based on the study data were estimated for SNPs where chromatin state assignment was listed. Estimation was performed using linear regression. Violation of linear regression assumption was considered if variance inflation factor > 10 and condition index > 30. Correlation between gene expressions was assessed using Spearman’s rank correlation.

**Table 6:** Primer sequences and accession numbers of target and reference genes.

Gene	Accession	Fw Primer 5′–3′	Rv Primer 5′–3′
*MMD*	NM_012329.3	TTGGTTTATCTGGCTCATGG	TGAAGTCCATCGGTGTTGTT
*ELOVL7*	NM_024930.3 NM_001104558.2 NM_001297617 NM_001297618.2	GCCAGCCTACCAGAAGTATT	CCTCCATGAAAAAGAACTGG
*ACTB*	NM_001101.3	CATCGAGCACGGCATCGTCA	TAGCACAGCCTGGATAGCAAC
*B2M*	NM_004048.2	TTCTGGCCTGGAGGCTATC	TCAGGAAATTTGACTTTCCATTC
